# Isometric points in cerclage for patellar tendon repair: a 3-dimensional, weightbearing computed tomography kinematic simulation

**DOI:** 10.1007/s00402-026-06441-x

**Published:** 2026-08-06

**Authors:** Christoph Zindel, Sandro Hodel, Yichao Luo, Michel Meisterhans, Sandro F. Fucentese, Lazaros Vlachopoulos

**Affiliations:** Department of Orthopedics, Balgrist University Hospital, University of Zurich, 8008 Zurich, Switzerland

**Keywords:** High tibial osteotomy, Posterior slope, Patientspecific instruments, 3D preoperative planning

## Abstract

**Purpose:**

The gold standard for the management of ruptured patellar tendon is the surgical repair with nonabsorbable sutures and additional reinforcement e.g. cerclage wires as described by McLaughlin in 1947. Despite the early description, the exact placement of such cerclage wires remains unclear. The aim of the study was (1) to analyze how the cerclage elongates during knee flexion and (2) to define the insertion points with the most isometric behavior at the patella and tibial tuberosity (TT).

**Methods:**

3D simulations of 10 healthy knees in the range from 0° to 120° of flexion using weightbearing computed tomography scans were generated. 12 insertion points were defined at the patella in equal distances along the lateral and medial border from the proximal to the distal pole. 42 insertion points were defined at the tibial tuberosity (TT) in a 3.0 × 3.0 cm large grid around its center. An automatic string simulation algorithm was used to calculate the length for all possible combinations of the cerclage from 0° to 120° flexion. The most isometric combination of insertion points was found using the isometric score.

**Results:**

The most isometric insertion at the TT is distally (*p* < 0.01) and towards the center of the TT (*p* < 0.01). The most isometric points at the TT is located 61 mm distal to the joint line and 5 mm from the anterior border of the TT in a true lateral view fluoroscopy. The insertion on the patella is insensible, as long as the patella is not bypassed proximally (*p* < 0.01).

**Conclusion:**

Elongation of patella tendon augmentation or cerclage can be reduced when choosing a distal and central insertion at the TT, while the insertion on the patella does not affect elongation as long as the patella is not by passed proximally. This might help to reduce excessive stress and prevent cerclage or augmentation failure.

## Introduction

Patellar tendon ruptures (PTR) are uncommon injuries that occur with an incidence of 0.68 per 100,000 person-years [7]. These injuries often happen in middle-aged individuals during activities that involve sudden quadriceps contraction with moderate knee flexion, such as sprinting or trying to avoid a fall [32]. Surgical treatment is typically recommended for complete PTR, with different techniques used, depending on the type of tear. For mid-substance tears, an end-to-end suture may be performed, while avulsion injuries from the tip of the patella may be repaired using transosseous sutures or suture anchors [15, 24, 29]. However, there have been reports of re-rupture rates as high as 7.5% ^29^. To achieve the best possible clinical outcome, many of the proposed repair techniques involve augmentation to protect the repair and allow early rehabilitation [8]. To augment the repair, various methods can be utilized, including cerclage wires, Dall-Miles cables, nonabsorbable sutures, autologous hamstring grafts, tendon allografts, and lately synthetic ligaments [4, 6, 8, 33]. Cerclage wires have shown high success rates, but they are associated with frequent complications such as material breakage [4, 6, 33], which may lead to inferior clinical results and a second surgical procedure to remove damaged material [3].

A reason for the material failure could be the misplaced cerclage wires. But despite the early description of the wire cerclage by McLaughlin et al. [28] in 1947, the exact placement of such cerclage wires remains unclear in the literature. McLaughlin et al. [28] recommended to place the cerclage wire just proxima1 to the patella and anchor to a bolt through the tibia1 tuberosity. The further development of the technique led to suggestions to place the cerclage wire in the patella through the mid-patellar axis [31] or in the distal pole of the patella [1, 22]. Regarding the wire anchorage in the tibia, the literature does not give more precise recommendations as placing the wire in the TT [1, 22, 28, 31]. No existing study in literature investigates the influence of the different wire positions to its biomechanical influence.

Incorrect placement of a cerclage or an augmentation in a non-isometric manner may cause excessive stretching or compression, subsequently leading to failure. Thus, it is crucial to understand how the cerclage elongates throughout the entire range of motion (ROM) and identify insertion points on the patella and tibia that minimize length changes.

The main objective of this study was (1) to analyze how the cerclage elongates during knee flexion using a weightbearing 3D computer tomography (CT) model. Additionally, (2) the study aimed to determine the insertion points on the patella and tibia that result in the smallest length change during knee flexion and report their location with respect to quantitative radiographic landmarks. We hypothesized that (1) the length change patterns depend on tibial and patellar insertion points and (2) that it is possible to identify insertion points at the patella and tibia exhibiting minimal length changes during knee flexion (referred to as isometric points).

## Materials and methods

The study was approved by the local ethical committee (Zurich Cantonal Ethics Commission, KEK-ZH-Nr 2013 − 0374) and all volunteers gave their informed consent.

A 3D simulation was performed using weightbearing CT data of 10 healthy volunteers. This data was obtained for a previous study [5]. The participants were at age of 35 years (range: 25–42 years), with a mean weight of 83 kg (range: 62–85 kg), and with a mean height of 180 cm (range, 169–190 cm). None of the volunteer reported neither previous knee pain nor knee surgery. The acquired high-resolution CT images were obtained in standing position with increasing knee flexion (0°, 30°, 60° and 120°) using an open extremity CT scanner (Verity, Planmed; slice thickness, 0.4 mm).

**Fig. 1:**
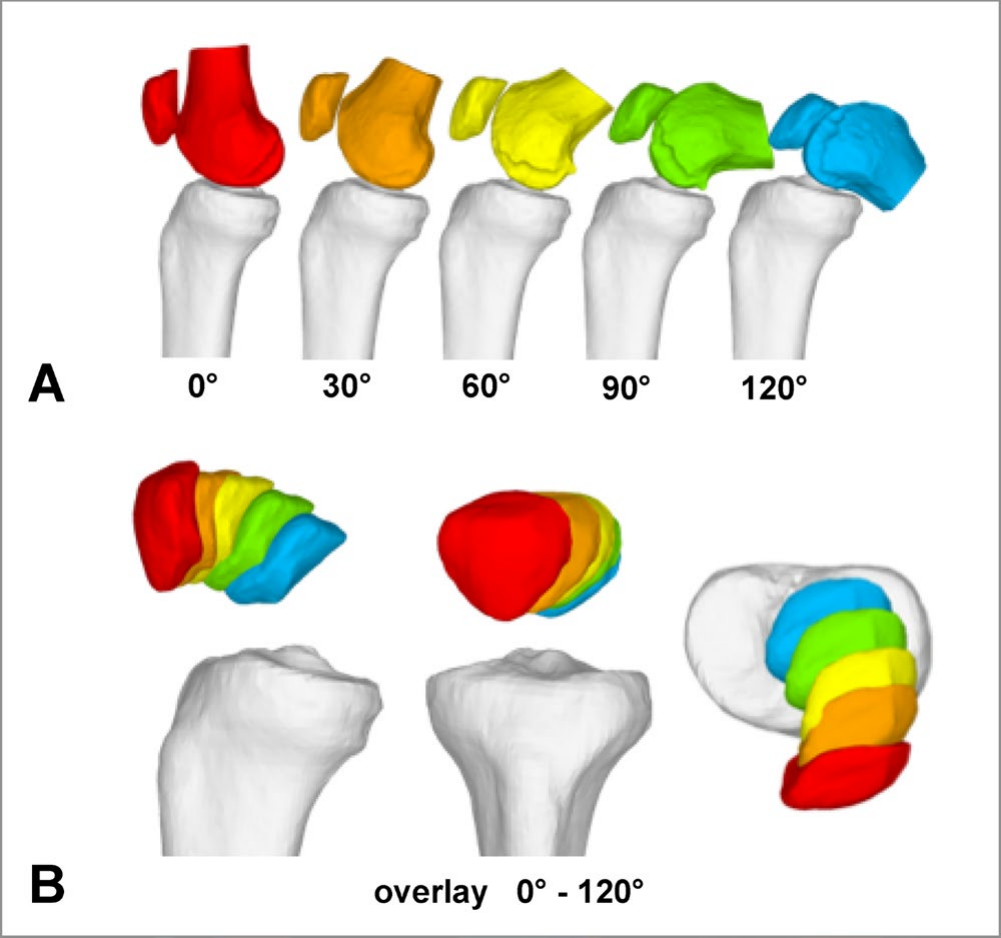
Simulation for full range of motion. The 3D bone models of the different stages of flexion (0° − 120°) are superimposed on the tibia to represent the relative movement of the patella. **A** shows the configuration during each stage of flexion over the full ROM. **B** shows an overlay of the patellae of all stages of flexion in lateral, frontal and axial view

This CT data allowed to perform image segmentation using Mimics software (Materialise) to reconstruct 3D triangular surface models [21, 23, 26, 27, 35]. The 3D models were postprocessed in the in-house 3D planning software (CASPA, Balgrist CARD AG, Zurich, Switzerland) [9, 10, 17, 18]. Similar to previous studies [19, 20], the tibia remained fixed as a reference, and the patella and femur movement was described relative to the tibia through-out flexion (see Fig. 1). The tibial model of each volunteer was superimposed using an iterative closest point (ICP) surface registration algorithm [2, 35]. To add the information at 90° flexion, the position of the patella was interpolated using the femora as references.

### Definition of the possible insertion points on the patella and TT

**Fig. 2:**
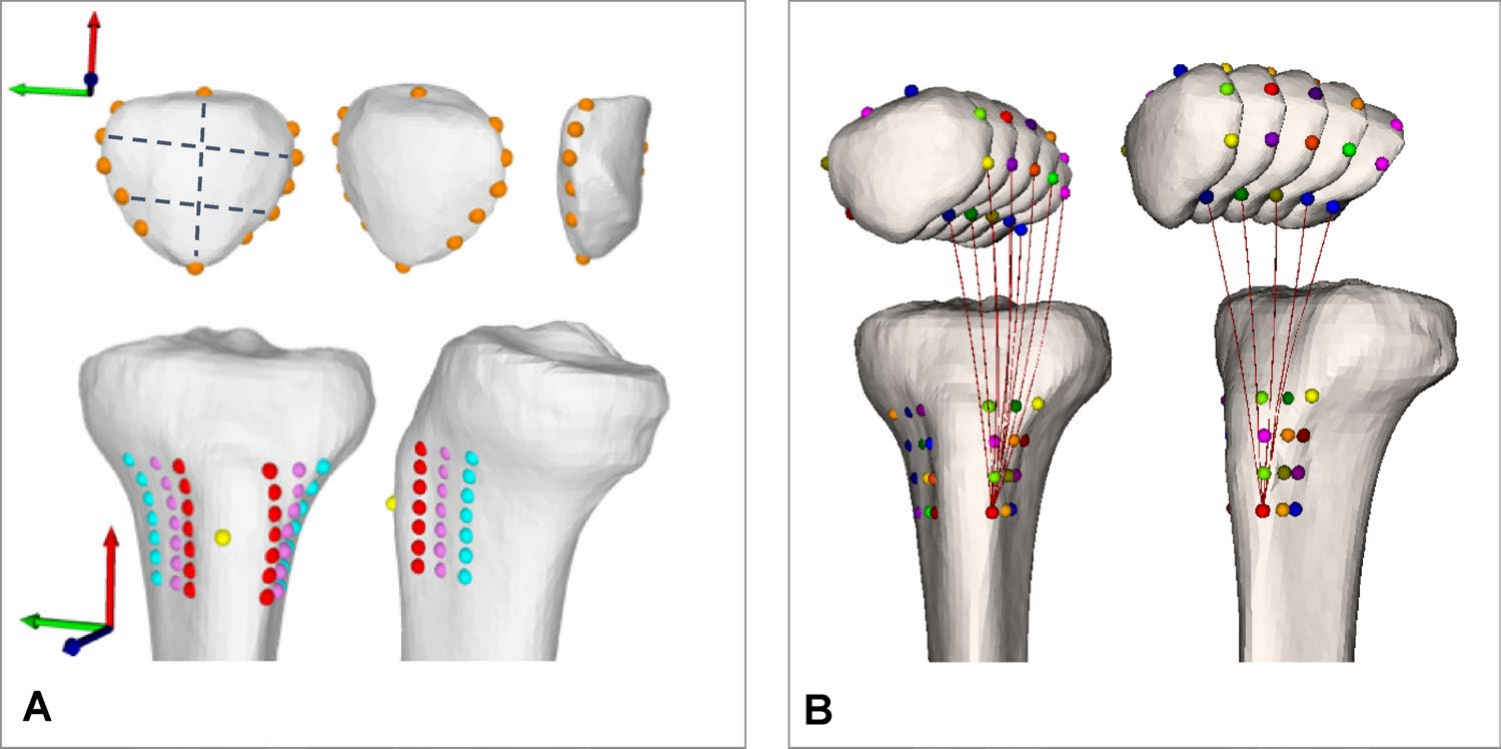
**A** Possible insertion points. On the Patella 12 insertion points were chosen divided in proximal pole (1 point), proximal sixth (2 points, 1 medial and 1 lateral), proximal third (2), middle (2), distal third (2), distal sixth (2) and distal pole (1). On the TT are 42 points (21 lateral and 21 medial in each 3 proximodistal rows (central (marked in red), middle (fuchsia), medial/lateral (turquoise)) of 7 points each). The tip of the TT is marked in yellow. The two coordinate systems indicating x (red), y (green) and z (blue) of the patellar respectively the tibial coordinate system. **B** Example of the simulated strings. The red lines simulate different possibilities of strings for a possible insertion point at the tibia and 10 respect. 5 points at the patella

The possible insertion points on the patella were chosen according to the literature [1, 22, 28, 31] to distinguish between the proximal, middle and distal third of the patella. Hence a coordinate system was set with a vector (x) connecting the distal and the proximal patella pole, a medial directing vector (y) and an anterior directing vector (z). In addition to the so resulting x-y-plane, 5 auxiliary planes were placed parallel to the z-y-plane, each at one sixth of the x-distance between the proximal and distal pole. Thereafter all 12 points were aligned along the outer circumference of the patella in the intersections with the x-y-plane and the auxiliary planes (see Fig. 2). It was visually confirmed that the defined points did not interfere with the articular surface of the patella.

The possible insertion points at the TT were defined on the bony surface in a grid around the tip of the TT (see Fig. 2). Therefore a coordinate system was set similar to Grood and Suntay [11] with a proximal directing vector parallel to the anatomical axis of the tibia (x) and a medial directing vector parallel to the posterior condyle axis (y) and an anterior directing vector (z). The tip of the TT was defined as the most anterior point in z-direction of the tuberosity. A medial and lateral grid was computed in the true medial respectively lateral view of the 3D CT model. The grid was defined as a rectangle with a proximodistal length of 30 mm and anterioposterior length of 15 mm each in 5 mm steps. The orientation of the grid was defined according to the x-z-plane and the anterior border respectively the proximodistal midline of the grid was defined by the tip of the tuberosity. Taking all grid points on the bony surface into account led to a total of 42 points (21 medially and 21 laterally) for the possible insertion points on the TT (see Fig. 2).

### Definition of string length, string length changes and the isometric score

Simulation for any type of cerclage or augmentation was conducted as a 3D string. The string was conducted individually for the lateral and the medial side. We examined the lateral and medial strings independently, under the assumption that the friction within the bony tunnels is too big that balancing between the medial and lateral sting would occur. Simulation of the string length was done using an automatic string generation algorithm based on the method described by Graf et al. [14]. It computed the length for all possible combinations of patellar and TT insertion points for all different stages of flexion. This resulted in a total of 1’470 data points.

The maximal length change of the string was defined as the elongation or shortening compared to the initial length at 0° flexion. Positive values indicated an elongation while negative values indicated a shortening during knee flexion.

The isometric score (*I*_*s*_) was used according to Graf et al. [14] resulting in one score throughout the chosen ROM (see Eq. 1). The isometric score was calculated by summing the relative mean square errors of all generated string lengths over a certain ROM. A score of 0 represents perfect isometry, while an score > 0 indicates increasing anisometry.

Isometric score (I_s_):

1 $$\:{I}_{S}=\frac{1}{\left|P\right|}\sum\:_{p\in\:P}^{c}{\left(\frac{{l}_{p,s}-{\stackrel{-}{l}}_{s}}{{\stackrel{-}{l}}_{s}}\right)}^{2}$$

where P is the set of all chosen flexion positions, l_p, s_ is the calculated length in flexion position p, l_s_ is the average length of the string over all flexion positions, and |P| is the number of measured flexion positions.

### Definition of the most isometric insertion points on the patella and TT

We aimed to find the string length combination that demonstrated the most isometric score throughout a full ROM (0° − 120°) and a reduced ROM (0° − 90°) for the medial and lateral side individually. The most isometric combination with its insertion points was reported as points on the patellar respectively on the TT grid. Furthermore, a subanalysis to differ between different regions on the patella and TT was conducted. Therefore the insertion points are grouped for the patella into bypassing proximally (though the quadriceps tendon along the proximal boarder of the patella), proximal third, middle third and distal third whereas for the TT into central, middle and lateral/medial (see coloring in Fig. 2).

In order to accurately identify the reported quantitative measurements in intraoperative fluoroscopy, we used manual annotation of anatomical landmarks that defined the tibial grid (e.g. the tip of TT) in the 3D model. We then created a digitally reconstructed radiograph, following the method described by Esfandiari et al. [12]. The quantitative radiographic landmarks were analyzed in a true lateral view, which was confirmed by ensuring that the medial and lateral tibial plateau overlapped congruently.

### Statistical analysis

The data were reported as means ± standard deviations (STD) and ranges. The isometric scores were analyzed using the Wilcoxon test. Two-tailed paired *t*-test was applied for subgroup analysis. The statistical analysis was performed using SPSS version 29 (SPSS Inc, Chicago, IL, USA).

## Results

All lateral string possibilities had an average length of 89.3 ± 14.6 mm (range 60.8 to 118.8 mm), whereas all medial string possibilities had an average length of 93.2 ± 13.9 mm (range 69.1 to 119.7 mm). The average maximal elongation was laterally 2.9 ± 2.6 mm (range 0.2 to 8.2 mm) and medially 2.7 ± 4.5 mm (range 0.0 to 13.7 mm) for the full ROM (0° − 120°), whereas the maximal shortening was laterally 1.4 ± 0.7 mm (range 0.6 to 3.8 mm) and medially 3.4 ± 2.4 mm (range 0.0 to 9.8 mm). This corresponds to an average elongation of 3.2% laterally and 2.9% medially and an average shortening of 1.5% laterally and 3.6% medially. Lengthening of the string occurs mainly lateral in 0° to 60° of knee flexion whereas shortening of the string occurred mainly medially over the full ROM and lateral in 60° to 120° of knee flexion.

### Isometric score and most isometric insertion points at patella and TT

Comparing the different groups on the patella (bypassing proximally, proximal third, middle third, distal third), only “bypassing proximally” (though the quadriceps tendon along the proximal boarder of the patella) showed significant differences to the other groups (more elongation, *p* > 0.05) in the isometric score. Otherwise no significant differences were found between the groups neither for the lateral (*p* = 0.375, *p* = 0.352, *p* = 0.212) nor for the medial side (*p* = 0.239, *p* = 0.101, *p* = 0.491).

The subgroup analysis for the TT demonstrated, for laterally as well as for medially, a significant lower isometric score for the distal group compared to the proximal (*p* < 0.05). The analysis showed as well a significant lower isometric score for the central group then the lateral (*p* < 0.05) respectively medial group (*p* < 0.05). Please refer to Table 1 for more details.

**Table 1:**
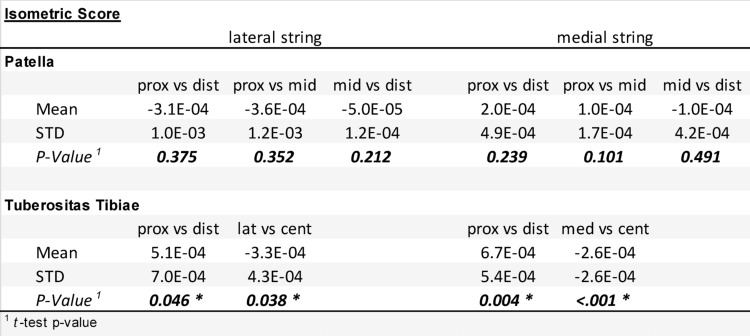
Subanalysis of isometric score for different groups of patella and TT

The isometric score of the lateral string was 1.28 ± 1.67 × 10^− 3^ and for the medial string 2.02 ± 2.98 × 10^− 3^ (*p* = 0.07) during full knee flexion of 0° to 120° in all participants. For the reduced ROM (0° − 90°), the isometric score of the lateral string was 6.13 ± 6.60 × 10^− 4^ and for the medial string 2.03 ± 3.46 × 10^− 3^. No statistically significant difference was observed between the full ROM (0° − 120°) and the reduced ROM (0° − 90°) for both the lateral (*p* = 0.293) and medial (*p* = 0.706) sides.

**Fig. 3:**
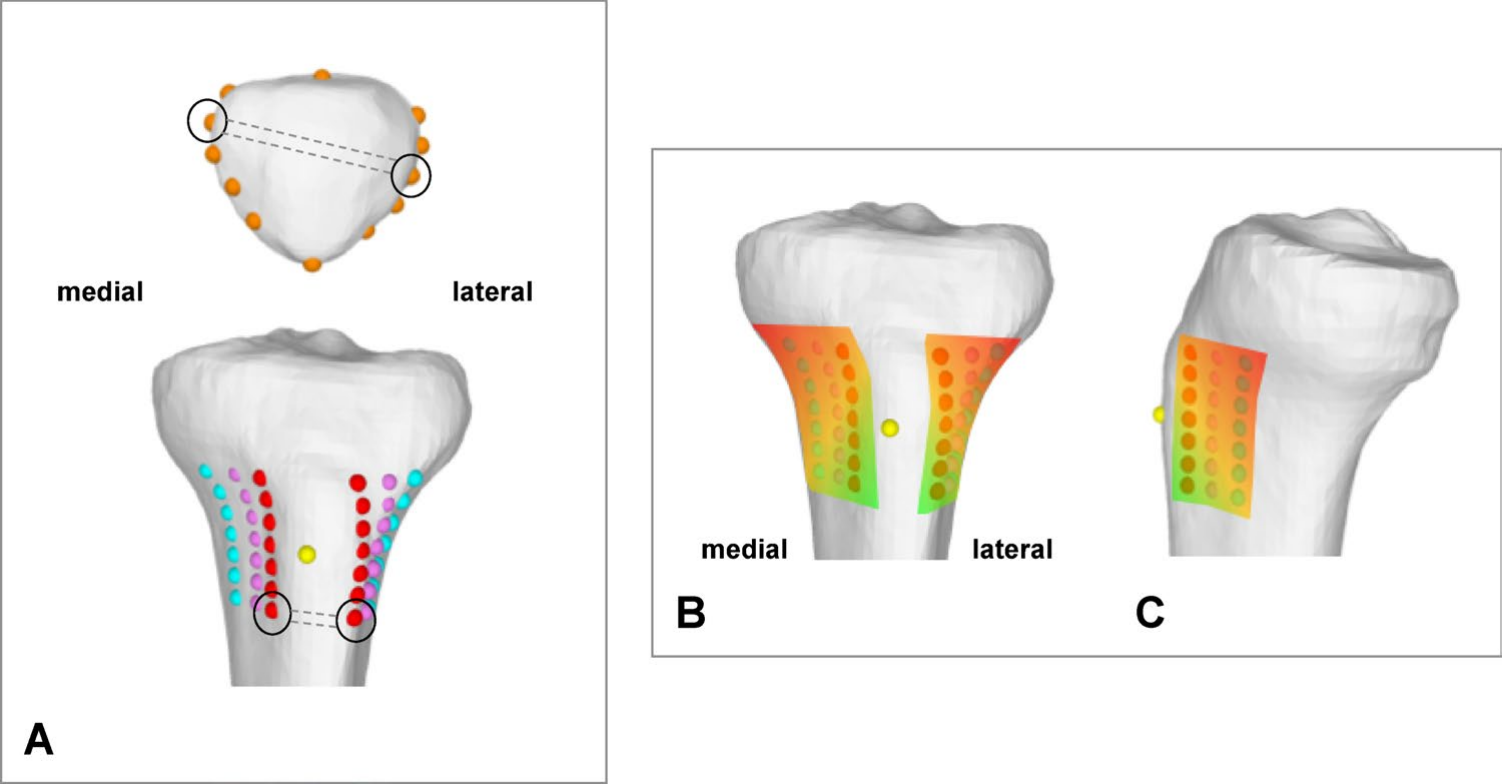
Isometric insertion points. **A** shows the most isometric insertion points laterally (green) und medially (blue) with a illustration of the possible string and its necessary drill holes through the patella and tibia. **B** and **C** shows a heat map with the most isometric insertion points in green and the least isometric in red in a frontal (**B**) and lateral (**C**) view

The combination of insertion points with the best isometric score laterally (*I*_*s*_ = 1.04 × 10^–4^) and medially (*I*_*s*_ = 2.41 × 10^–4^) are marked in Fig. 3. In a true lateral view of the digitally reconstructed radiograph, these points on the TT could be identified 61 mm distal to the joint line (equivalent to 15 mm distal to the tip of the TT) and 5 mm posterior of the anterior tibial edge medially respect. lateraly. The most isometric point on the patella would be found in the middle of the proximodistal axis for the lateral insertion point and in the proximal third for the medial insertion point, however, not yielding significance between the groups (proximal, middle, distal).

If choosing the combination of insertion points with the best isometric score, the average string length is lateral 97.5 ± 74. mm (range 87.6 to 109.9 mm) and medial 94.6 ± 5.9 mm (range 86.4 to 103.1 mm) in all participants. The average maximal elongation was laterally 1.7 ± 1.7 mm (range 0.0 to 5.3 mm) and medially 0.5 ± 0.7 mm (range 0.0 to 2.1 mm) for the full ROM (0° − 120°), whereas the maximal shortening laterally 0.8 ± 1.5 mm (range 0.0 to 4.8 mm) and medially 3.8 ± 3.0 mm (range 0.0 to 9.3 mm) was. This corresponds to a mean maximal elongation of 1.7% laterally and 4.0% shortening medially.

## Discussion

The most important finding of this study is, that choosing the insertion point at the most isometric points leads to a significant lesser change in length of the string on the lateral and medial side over the full ROM. This confirms our second hypothesis and can potentially reduce the stress on the wire material and, consequently, decrease the likelihood of material breakage. This is important as material breakage is a frequent complication associated with wire cerclage [4, 6, 33] and can lead to failure of the PTR with inferior clinical outcomes and the need for additional surgical procedures to revise the PTR and/or remove damaged material [3]. Using the most isometric insertion points may also result in less overconstraining and less overtightening when employing the suture tape technique [13], potentially minimizing the occurrence of patellofemoral chondrosis [25]. This as well can lead to a more functional rehabilitation with a greater tension-free ROM. Here is to mention, that tightening the cerclage should be carried out in slight knee flexion and with slight tension on the patellar tendon. In full knee extension, the patellar tendon might be relaxed and the cerclage tightened too tightly as a result.

We successfully identified the most isometric insertion points on the TT and patella. Our subgroup analysis showed that the length change is significantly lower when choosing a more central and distal insertion point on the TT. However, for the patella, we found no significant differences as long as the string does not bypass the patella proximally from the medial side. The literature does not provide specific information about the insertion points on the TT [1, 22, 28, 31], so we cannot compare our findings to existing studies. Regarding the insertion points on the patella, the literature is divided on whether it is best to pass through the proximal [28], middle [31], or distal third [1, 22] of the patella. Our study supports all of these recommendations, as we did not find any significant differences. Therefore, other factors such as concomitant injuries, surgical approach or the thickness of the patella can be taken into consideration when selecting the insertion points on the patella.

We were as well able to confirm our first hypothesis, as the analysis of the length over the different stages of the flexion found that the string lengthens primarily lateral in 0° to 60° of knee flexion while it shortens mainly medially over the full ROM and lateral in 60° to 120° of knee flexion. This is most likely because of the anteroposterior translation of the lateral knee compartment and the resulting relative internal rotation of the tibia during weightbearing [30] (see Fig. 1 – part B). The study did not evaluate the elongation of the string in non-weightbearing situations, so we cannot provide any conclusions or statements regarding its behavior in those scenarios.

In previous described surgical procedures for PTR reconstruction, intraoperative fluoroscopy was not commonly used. However, recent literature [8, 16, 34] suggests that utilizing fluoroscopy during surgery can have significant benefits for clinical outcomes, particularly when attempting to reproduce the Caton-Deschamps Index from the unaffected side. In cases of acute injury, identifying the native anatomical landmarks can be challenging, but intraoperative fluoroscopy can aid in reproducing radiographic landmarks accurately. Therefore, the reported quantitative radiographic landmarks for determining the most isometric insertion points can also be valuable in guiding surgical reconstruction of the patellar tendon. According to the study findings, the most isometric insertion point on the tibia is located approximately 61 mm distal to the joint line and 5 mm posterior to the anterior edge of the tibia.

### Limitations

There are several limitations that should be taken into consideration when interpreting the findings of this study. Firstly, the sample size of only 10 participants is relatively small, which may limit the generalizability of the results. Additionally, the use of a mean tibial model may not account for individual variations in anatomy. Further, the string generation algorithm used in the study simplifies the course of the string and does not consider factors such as soft tissue (e.g. ligaments, joint capsule) or biomechanical properties like wire twist.

## Conclusions

Elongation of patella tendon augmentation or cerclage can be reduced when choosing a distal and central insertion at the TT, while the insertion on the patella does not affect elongation as long as the patella is not by passed proximally. This might help to reduce excessive stress and prevent cerclage or augmentation failure.

### What is known about the subject

There are different recommendations about the ideal insertion points at the patella and TT when applying a cerclage wire as reinforcement while doing a surgical repair of a patellar tendon rupture. McLaughlin et al. recommended to place the cerclage wire just proxima1 to the patella. Further clinical studies led to the suggestions to place the cerclage wire in the patella through the mid-patellar axis or in the distal pole of the patella. But there exists no study so far analyzing the biomechanics of the wire cerclage in 2D or 3D so far.

### What this study adds to existing knowledge

Our study shows the first time an analysis of the biomechanics of the application of a wire cerclage in surgical repair of a patellar tendon rupture. It could be shown that elongation of patella tendon augmentation or cerclage can be reduced when choosing a distal and central insertion at the TT, while the insertion on the patella does not affect elongation as long as the patella is not by passed proximally. This groundbreaking insight offers a pivotal advancement in surgical techniques for patellar tendon repair, addressing a critical gap in the existing literature.

## Data Availability

No datasets were generated or analysed during the current study.

## Declarations

### Clinical relevance

TThe knowledge of the isometric points while applying a cerclage for patellar tendon repair can drastically reduce the material failure and therefore the early removal of the cerclage.
